# Socioeconomic differences in perinatal health outcomes: perinatal health surveillance through a health-equity prism

**DOI:** 10.1093/eurpub/ckac129.111

**Published:** 2022-10-25

**Authors:** L Smith, A Farr, O Zurriaga, M Cuttini, I Verdenik, MJ Vidal Benedé, K Kearns, L Sakkeus, T Kyprianou, H Barros

**Affiliations:** 1 Department of Health Sciences, University of Leicester, Leicester, UK; 2 Department of Obstetrics and Gynecology, Medical University of Vienna, Vienna, Austria; 3 Public Health and Preventive Medicine Department, University of Valencia, Valencia, Spain; 4 Clinical Care and Management Innovation Research Area, Bambino Gesù Children’s Hospital, Rome, Italy; 5 Department of Obstetrics and Gynecology, University Medical Centre, Ljubljana, Slovenia; 6 Public Health Agency of Catalonia, Barcelona, Spain; 7 Healthcare Pricing Office, National Finance Division, HSE, Dublin, Ireland; 8 Estonian Institute for Population Studies, Tallinn University, Tallinn, Estonia; 9 Health Monitoring Unit, Ministry of Health, Nicosia, Cyprus; 10 EPIUnit, Instituto de Saúde Pública, University of Porto, Porto, Portugal

## Abstract

**Background:**

Socioeconomic status (SES) is strongly associated with perinatal health outcomes, perpetuating intergenerational health inequalities. Our aim was to assess the utility of population data in Europe to monitor social inequalities in key perinatal health indicators.

**Methods:**

Using the PHIRI federated analysis protocol to aggregate routine birth data from across Europe, we collected data on selected perinatal health indicators by SES from 2015 to 2020. Mothers’ education level (primary/lower secondary; upper secondary; postsecondary) was the preferred SES indicator; if unavailable, parents’ occupation or area-based deprivation scores were provided. The International Standard Classification of Occupations was used to group parents’ occupations into 4 categories, while area-based deprivation scores were measured in quintiles. For each country, we calculated risk ratios (RR) for preterm birth, stillbirth, neonatal death and caesarean delivery (CD) comparing the most with the least disadvantaged group

**Results:**

17 countries provided data on maternal education, 5 on area-based deprivation, 1 on parents’ occupation and 2 could not provide data. For preterm birth, stillbirth and neonatal death, lower SES was associated with worse outcomes with most RR between lowest and highest groups in the range of 1.5 to 3.0. In contrast, in some countries, such as Croatia, Latvia, Lithuania and Spain, CD rates were higher for socially advantaged groups whereas the gradient was reversed in others (Denmark, Luxembourg, the Netherlands and Italy).

**Conclusions:**

European countries can provide perinatal health indicators by SES, revealing marked socioeconomic inequalities in perinatal health. The differing SES gradient between countries for CD raise questions about care organization and clinical practice. Further exploration of the harmonization of differing SES measure across countries is required, while countries that do not monitor SES data should aim to improve existing systems.

